# *SF3B1* mutation is a prognostic factor in chronic lymphocytic leukemia: a meta-analysis

**DOI:** 10.18632/oncotarget.19455

**Published:** 2017-07-22

**Authors:** Zhenghao Zhang, Shu Chen, Shuang Chen, Gang Chen, Rui Zhang, Jinhua Li, Jianhua Qu

**Affiliations:** ^1^ Centre of Hematology, The First Affiliated Hospital of Xinjiang Medical University, Urumqi 830054, China; ^2^ Department of Pathology, The First Affiliated Hospital of Xinjiang Medical University, Urumqi 830054, China

**Keywords:** SF3B1, chronic lymphocytic leukemia, prognosis, survival, meta-analysis

## Abstract

Recent studies suggest that *SF3B1* might be related to poor prognosis in CLL, but the results remain controversial. This meta-analysis was performed to clarify the relationship between *SF3B1* mutation and prognosis in patients with CLL. The relevant published reports were searched in PubMed, EMBASE, and Web of Science. A total of 13 articles were included in this meta-analysis as they met the inclusion and exclusion criteria. The hazard ratios (HRs) and corresponding 95% confidence intervals (95%CIs) for progression free survival (PFS) and/or overall survival (OS) were extracted from each eligible study. The pooled HR evaluating *SF3B1* mutation on PFS was 1.81(95%CI 1.33-2.46, I^2^=78.9%, P<0.001) and on on OS was 2.57(95%CI 1.68-3.94, I^2^=79.3%, P<0.001) by random effects model. In conclusion, *SF3B1* mutation was significantly associated with poor PFS and OS in CLL. It could be consider as a potential prognostic factor in patients with CLL.

## INTRODUCTION

Chronic lymphocytic leukemia (CLL) is a clinical and biological heterogeneous malignant disease characterized by an accumulation of monoclonal CD19^+^CD5^+^CD23^+^ mature small B-lymphocytes in bone marrow, blood, and lymphoid tissues [1]. Some patients show an indolent clinical course without requiring special therapy, while others having an aggressive course and short survival despite following intensive treatment [2–4]. Therefore, It is very important for us to identify prognostic factors, which could precisely predict the survival and disease progression and choose the reasonable treatment and preventive measures for patients with CLL [5–7]. Some of the cytogenetic and molecular biomarkers, such as *TP53* disruption and immunoglobulin heavy chain variable region (IGHV) mutational status, *CD38* and *ZAP-70* expression, have been proved to be associated with the prognosis of CLL [8–12]. Even with these biomarkers, however, prediction of the survival and disease progression is not highly reliable.

Splicing factor 3B subunit 1 (*SF3B1*) is locate on chromosome 2q33.1. The cDNA is 43074 bp long and comprises 25 exons. The *SF3B1* protein is part of the spliceosome machinery and plays an important effect of RNA splicing [13]. In western countries, *SF3B1* mutation was detected in about 5-18% of newly diagnosed CLL patients [14–15]. Recent data suggest that *SF3B1* has been reported as one of the prognostic markers in CLL [16–18]. However, the prognosis of *SF3B1* mutation in CLL patients was still controversial. Some studies showed that *SF3B1* mutation have been associated with a relatively poor prognosis, whereas other studies suggested no significant prognostic value in CLL [19, 24]. Thus, it is necessary to perform a meta-analysis to further clarify the relationship between *SF3B1* mutation and prognosis in patients with CLL.

## RESULTS

### Selection and characteristics of the studies

**Figure 1:**
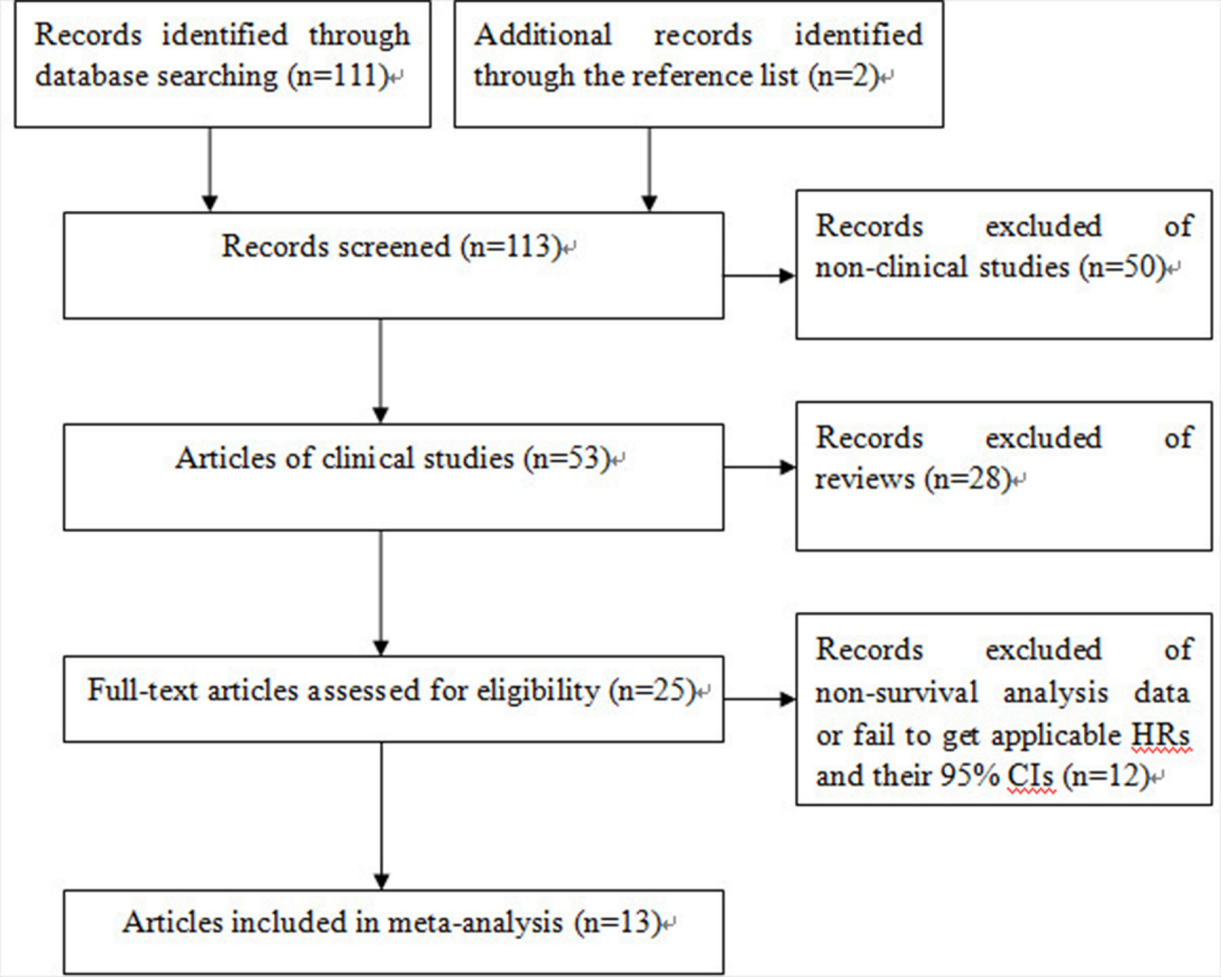
Study flow diagram of included studies

Figure 1 showed a flow diagram of the literature selection process. Through the initial database search, a total of 113 studies were included for detailed screening. After excluding non-clinical studies and reviews by reading titles and abstracts, 25 articles were considered potentially eligible and were retrieved in full text. After having excluded of non-survival analysis data or fail to get applicable hazard ratios(HRs) and their 95% confidence intervals(95% CIs), 13 articles [16, 19–30] were included in this meta-analysis as they provided at least one of the survival data, progression free survival (PFS) or overall survival (OS). Characteristics of the eligible studies were shown in Table 1.

**Table 1:** Characteristics of the eligible studies

First author	year	Patients source	Mutated N (PFS/OS)	Wild-type N (PFS/OS)	Methods	Outcomes	Data extraction
Nadeu F	2016	Spain	51/50	350/343	NGS	OS/PFS	Kaplan-Meier curve
Rossi D	2011	Italy	17/17	284/284	SS, NGS	OS/PFS	Direct (M)
Oscier DG	2013	United Kingdom	73/73	364/364	HRMA	OS/PFS	Direct (M)
Dreger P	2013	Germany	26/26	74/74	DHPLC, SS	OS/PFS	Direct (M)
Hurtado AM	2015	Spain	10/10	170/170	NGS	OS/PFS	Direct (M)
Stilgenbauer S	2014	Germany	114/NA	507/NA	DHPLC, SS	PFS	Direct (M)
Mitsui T	2016	Japan	NA/7	NA/80	SS	OS	Direct (M)
Jeromin S	2014	Germany	80/80	841/841	SS, NGS	OS/PFS	Direct (M)
Wang LL	2011	USA	14/NA	77/NA	NGS	PFS	Direct (M)
Schnaiter A	2013	Germany	17/17	77/77	DHPLC, SS	OS/PFS	Kaplan-Meier curve
Quesada V	2012	Spain	10/10	95/95	NGS	OS/PFS	Kaplan-Meier curve
Xia Y	2014	China	10/15	244/292	SS	OS/PFS	Kaplan-Meier curve
Rossi D	2013	Italy	NA/41	NA/542	SS, NGS	OS	Direct (M)

NGS: next generation sequencing SS: Sanger sequencing HRMA: high-resolution melt analysis DHPLC: denaturing high performance liquid chromatography NA: not applicable.

### Association between *SF3B1* mutation and OS/PFS of CLL in total population

**Figure 2:**
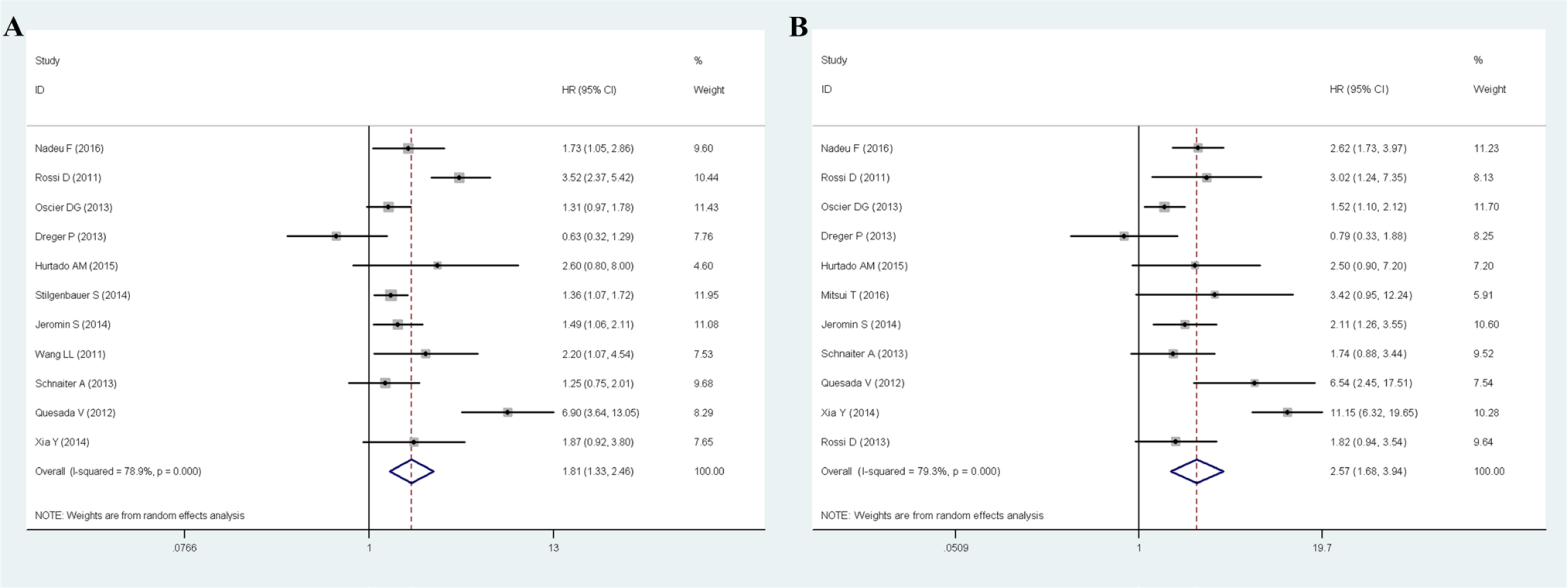
Meta-analysis of the association between SF3B1 mutation and PFS **(A)** / OS **(B)** of CLL.

The meta-analysis results of PFS of CLL were shown in Figure 2a. A total of 11 studies with available HRs and their 95% CIs for PFS have been included in the meta-analysis, with a total of 3505 CLL patients, 422 patients were mutant *SF3B1* and other 3083 patients were wild-type *SF3B1*. Overall, the pooled HR evaluating *SF3B1* mutation on PFS was 1.81(95%CI 1.33-2.46, *P*<0.001) by random effects model for the existence of a significant heterogeneity(I^2^=78.9%, *P*<0.001), which suggested that *SF3B1* mutation was significantly associated with worse prognosis for CLL in PFS (Figure 2A).

The meta-analysis results of OS of CLL were shown in Figure 2b. 11 studies with available HRs and their 95% CIs for OS have been included in the meta-analysis, with a total of 3175 CLL patients, 346 patients were mutant *SF3B1* and other 2829 patients were wild-type *SF3B1*. For the existence of a significant heterogeneity(I^2^=79.3%, *P*<0.001), random effects model were used in the analysis. Overall, the pooled HR for the 11 eligible studies evaluating *SF3B1* mutation on OS was 2.57(95%CI 1.68-3.94, *P*<0.001), which suggested that *SF3B1* mutation was significantly associated with worse prognosis for CLL in OS (Figure 2B).

### Prognostic impact of *SF3B1* mutation in different subgroups

For the existence of a significant heterogeneity, the HRs for PFS and OS were pooled in different groups by random effects model. To explain the heterogeneity, we performed a subgroup analysis in patients source, sample number, method and data extraction (Table 2, Supplementary Figures 1 -4). In the analysis of PFS, a part of the heterogeneity could be explained by sample number and detection methods, and in the analysis of OS, a part of the heterogeneity could be explained by patients source, sample number, detection methods and data extraction methods. In addition, there was no statistically significant association between the prognosis of CLL and *SF3B1* mutation in small sample size subgroup, PFS HR=0.93(0.48-1.81) and OS HR=1.53(0.74-3.18), and this also could be found in PFS of PCR-based methods group. Besides, we still observed a significant adverse impact of *SF3B1* mutation on PFS and OS in other subgroups.

**Table 2:** Subgroup analysis of association between SF3B1 expression and prognosis of CLL

	PFS				OS			
	No.	HR(95%CI)	P	I^2^(%)	No.	HR(95%CI)	P	I^2^(%)
**Patients source**								
Caucasian	10	1.81(1.30-2.51)	<0.001	80.9	9	2.05(1.53-2.75)	0.050	48.4
Asia	1	**1.87 (0.92-3.80)**	___	___	2	7.14(2.32-21.93)	0.098	63.5
**Sample size**								
≤100	2	**0.93(0.48-1.81)**	0.116	59.6	3	**1.53(0.74-3.18)**	0.145	48.3
>100	9	2.08(1.49-2.90)	<0.001	79.1	8	3.01(1.82-4.96)	<0.001	82.8
**Detection methods**								
Sequencing-based	7	2.49(1.63-3.80)	0.001	74.1	8	3.40(2.08-5.55)	<0.001	74.1
PCR-based	4	**1.24(0.99-1.54)**	0.239	28.9	3	1.44(1.05-1.97)	0.329	10.1
**Data extraction**								
Direct	7	1.61(1.15-2.26)	<0.001	76.6	7	1.79(1.36-2.36)	0.287	18.7
Kaplan-Meier curve	4	2.26(1.11-4.60)	<0.001	83.6	4	4.20(1.78-9.92)	<0.001	87.0

### Sensitivity analysis

**Figure 3:**
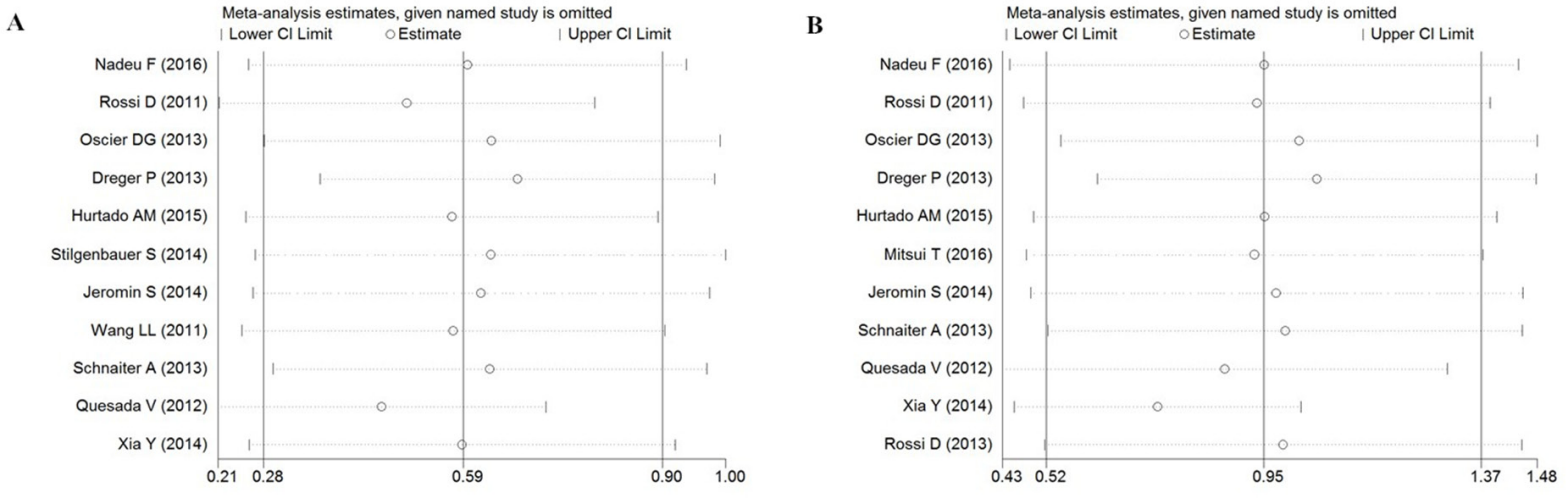
Sensitivity analysis of the association between SF3B1 mutation and PFS **(A)** / OS **(B)** of CLL.

We performed sensitivity analysis by sequentially excluding studies to verify the stability of results. The data about PFS and OS of total studies was used to do sensitive analysis. As shown in Figure 3, regarding the sensitivity analysis, the influence of each study on the pooled HR in CLL patients was examined by repeating the meta-analysis, while excluding a single study at a time. As for the association of *SF3B1* mutation with the worse prognosis of patients with CLL, the sensitivity analysis suggested that no individual study significantly altered the pooled HR.

### Publication bias

**Figure 4:**
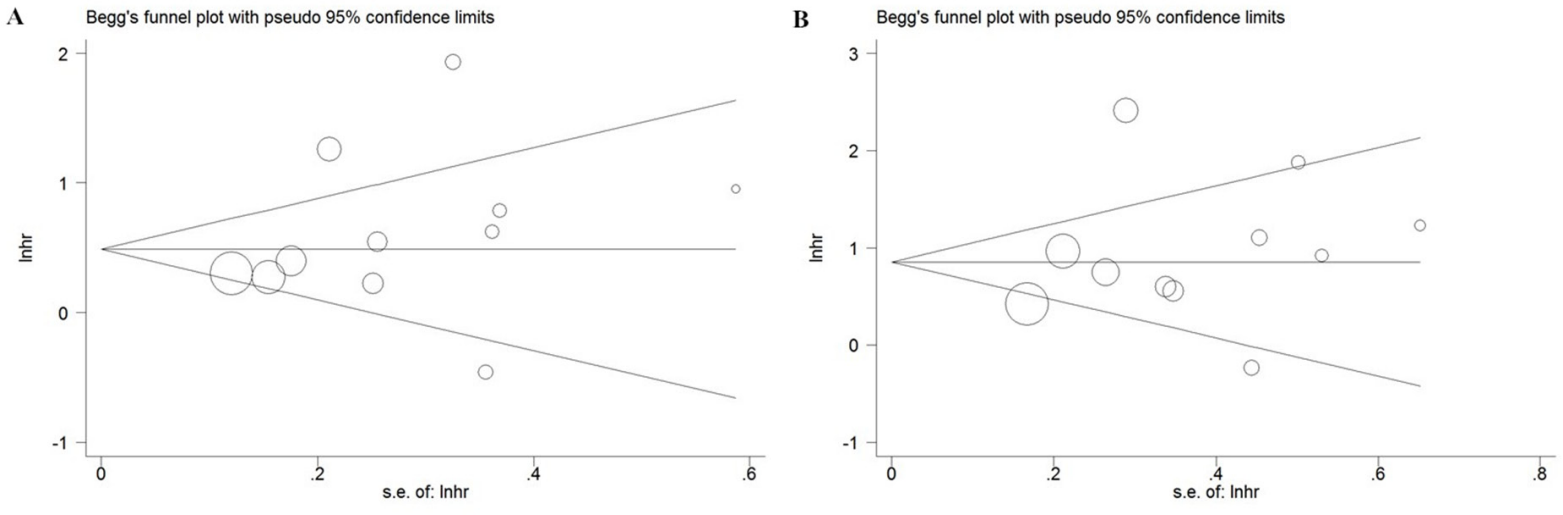
Funnel plot of the association between SF3B1 mutation and PFS **(A)** / OS **(B)** of CLL.

Begg's test and Begg’s funnel plot were used for assessment of publication bias in the meta-analysis. As shown in Figure 4, Visual inspection of the Begg’s funnel plots of PFS and OS were no substantial asymmetry. The Begg’s test for publication bias in the meta-analysis demonstrated no significant publication bias.

## DISCUSSION

With the development of next-generation sequencing technology, more and more biomarkers have been found to play a key role in diagnosis and prognosis of malignancy [31]. In 2011, Wang et al. performed next-generation sequencing of whole exomes and whole genomes on 91 patients with chronic lymphocytic leukemia. Nine genes that were mutated at significant frequencies were identified. Five of the genes with significant mutation frequencies had not be reported in CLL. Among these genes, *SF3B1* was the second most frequently mutated gene with mutations occurring in 15% of patients [16]. After this, more researches have researched the prognostic significance of *SF3B1* mutation in CLL patients. Some of them suggested that *SF3B1* mutation were associated with a poor prognosis, but the results were still conflicting and heterogeneous [19, 24, 28]. To our knowledge, our present study is the first meta-analysis to investigate the association between *SF3B1* mutation and prognosis in patients with CLL. we believe that our analysis will provide useful information for decision-making in CLL clinically.

The meta analysis combined 13 publications including 3505 patients for PFS and 3175 patients for OS, indicating the correlation between *SF3B1* mutation and prognosis of CLL. The overall pooled HR revealed that *SF3B1* mutation was associated with poor prognosis on PFS (HR=1.81, 95%CI 1.33-2.46) and OS (HR=2.57, 95%CI 1.68-3.94) in patients with CLL. SF3B1 is a critical component of both major and minor spliceosomes, which enact the precise excision of introns from pre-mRNA. The biological mechanism of SF3B1 mutation in CLL is unclear. Recent studies suggested that SF3B1 could control cell-cycle and apoptosis by alter the splicing. SF3B1 mutations lead to mistakes in the splicing of some specific transcripts that affect the pathogenesis of chronic lymphocytic leukemia.

However, a significant heterogeneity of the included studies was observed in this meta-analysis, so we used subgroup analysis and sensitivity analysis to elucidate the source of heterogeneity. We made the subgroup analysis about patients source for the possible heterogeneity of patients from different regions. Most of the studies were from European countries, only 2 studies were from Asia, 1 from the USA. In this subgroup analysis, the studies from European countries and USA were merged into one group. A significant correlation of *SF3B1* mutation with PFS and OS was observed in the caucasian subgroup. In the subgroup analysis of PFS, only one study was divided into Asia group (HR 1.87, 95%CI 0.92-3.80), which suggested that there was no statistically significant associations between *SF3B1* mutation and PFS of CLL patients. But *SF3B1* mutation was significantly correlated with poor OS of CLL in Asia (HR 7.14, 95%CI 2.32-21.93). This discrepancy might be caused by less studies of Asian, so more studies should further be needed in CLL patients of Asia.

Subgroup analysis indicated that sample size might account for part of included studies heterogeneity. Moreover, subgroup analysis indicated that *SF3B1* mutation was significantly correlated with poor PFS and OS of patients in subgroup of sample size >100 while not in subgroup of sample size ≤100. *SF3B1* mutation patients in studies of sample size ≤100 were too few, which could be a part of the reason for the discrepancy.

Furthermore, *SF3B1* mutation detection was performed by using one or more of the following methods: high-resolution melt analysis, denaturing high performance liquid chromatography, Sanger sequencing, or next generation sequencing. The sensitivity for the individual methods were different, it could be one of potential sources resulting in heterogeneity of included studies. According to the basic theory of detection methods, we divided the studies into two subgroups: sequencing-based and PCR-based. The subgroup analysis indicated that detection methods might also account for part of the heterogeneity, and the pooled results form PCR-based methods showed *SF3B1* mutation was no significantly correlated with PFS, but this could not be observed in OS.

If HRs and their corresponding CIs were not directly reported in the included studies, the available survival data was extracted from survival curve by using the data extraction software. In order to find the possible heterogeneity of patients from different data extraction methods, we also made the subgroup analysis about data extraction methods. Subgroup analysis indicated that data extraction might also account for part of the heterogeneity. Study results of the survival data were reported by multivariate and univariate analysis. In this study, all of the data of direct extraction was collected from multivariate analysis. In addition to these reasons, heterogeneity might derive from the patients with different clinical stage and type of treatments, due to the limitation of reported data [32, 33], we had not made a detailed analysis in this meta-analysis.

In sensitivity analysis, omission of any individual study did not reduce the heterogeneity or help to elucidate the source of heterogeneity [34]. A single study involved in the meta-analysis was deleted each time to reflect the influence of the individual data set to the pooled HR, and the corresponding pooled HR were not materially altered.

Publication bias is another important factor to be considered for all forms of meta-analysis [35]. The Begg’s tests and funnel plots for publication bias in the meta-analysis indicated no significant publication bias. However, it should be noticed that some of limitations should be recognized for our meta-analysis. For example, the analysis was limited in fully published studies in English, which probably introduced bias.

In conclusion, this meta-analysis indicated that *SF3B1* mutation was significantly associated with poor PFS and OS in CLL. It suggests that *SF3B1* mutation might be a predictive factor of poor prognosis in patients with CLL. However, more prospective studies with better standardized methods are needed to further confirm the results in this study.

## MATERIALS AND METHODS

### Publication search

The relevant published reports were searched in PubMed, EMBASE, and Web of Science updated to October 2016, with the following terms to search the databases: (“*SF3B1*” OR ‘‘Splicing factor 3B subunit 1’’) AND (‘‘Chronic lymphocytic leukemia’’ OR ‘‘CLL’’). Publication language was restricted to English in this study. Additionally, we also manually examined the reference lists of identified studies for potentially relevant papers.

### Study selection

Two reviewers(ZZ and SC) independently screened the titles and abstracts of articles identified by the literature search, retrieved potentially relevant studies and determined study eligibility. Eligible studies should include the following criteria: (1) the study evaluated the association between *SF3B1* mutation and the prognosis of patients with CLL; (2) the biomarkers of the study included *SF3B1*; (3) the *SF3B1* mutation of the study was reported with hazard ratios (HRs) and corresponding 95% confidence intervals (95%CIs) or with Kaplan-Meier plots; (4) the outcome was progression free survival (PFS) or overall survival (OS). Data published only in abstract form were excluded. Review articles, commentary articles, and studies with insufficient information for data extraction were also excluded.

### Data extraction

The following data including: the first author, year, source of the study population, mutated and wild-type number of patients included in analysis, detection methods, data extraction methods and HRs with their 95% CIs for OS and/or PFS were extracted from each eligible study by two investigators. If HRs and their 95% CIs were not directly reported in the included studies, but the survival curves of *SF3B1* mutation were presented, we extracted HRs and their 95% CIs from Kaplan-Meier curves by using a method reported by Tierney et al [36].

### Statistical analysis

For the quantitative aggregation of the survival results, HRs and their 95% CIs were used to measure the impact of *SF3B1* mutation on survival of CLL. HRs and their 95% CIs of the studies were pooled by using Stata software (version 12.0). Heterogeneity of studies was calculated by using Q test and I^2^ statistic. If P_Q_ > 0.10 or I^2^ < 50%, it meant there was no significant heterogeneity among the studies, and the pooled HR was combined by fixed-effect model. Otherwise, random-effect model was used [37]. By convention, an observed HR>1 implied a worse prognosis for the *SF3B1* mutation group. Otherwise, an observed HR<1 implied a better prognosis for *SF3B1* mutation group. The impact of *SF3B1* mutation on survival of CLL was considered to be statistically significant if the 95% CI did not overlap with 1. Sources of heterogeneity were analyzed by subgroup analysis. Sensitivity analysis was performed by sequentially excluding each study in turn to test the stability of the main results. The publication bias in the meta-analysis was assessed by Begg’s test and funnel plot. P < 0.05 was considered significant for the tests.

## SUPPLEMENTARY MATERIALS FIGURES


