# Supplementary material for: SF3B1 mutation is a prognostic factor in chronic lymphocytic leukemia: a meta-analysis
Source: Oncotarget. 2017 Jul 22;8(41):69916–23. doi: 10.18632/oncotarget.19455 (PMC5642526; doi:10.18632/oncotarget.19455)
Supplement: Supplementary file 1 [file oncotarget-08-69916-s001.pdf]

SF3B1 mutation is a prognostic factor in chronic lymphocytic leukemia: a meta-analysis

SUPPLEMENTARY MATERIALS

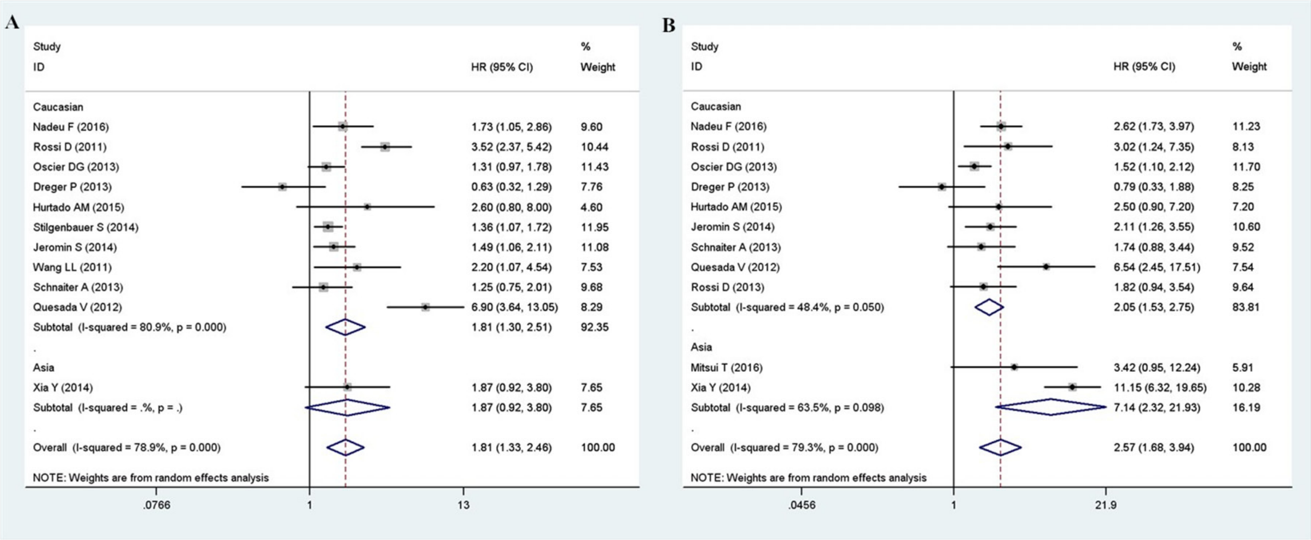

Supplementary Figure 1: Patients source subgroup analysis of the association between SF3B1 mutation and PFS **(A)** / OS **(B)** of CLL.

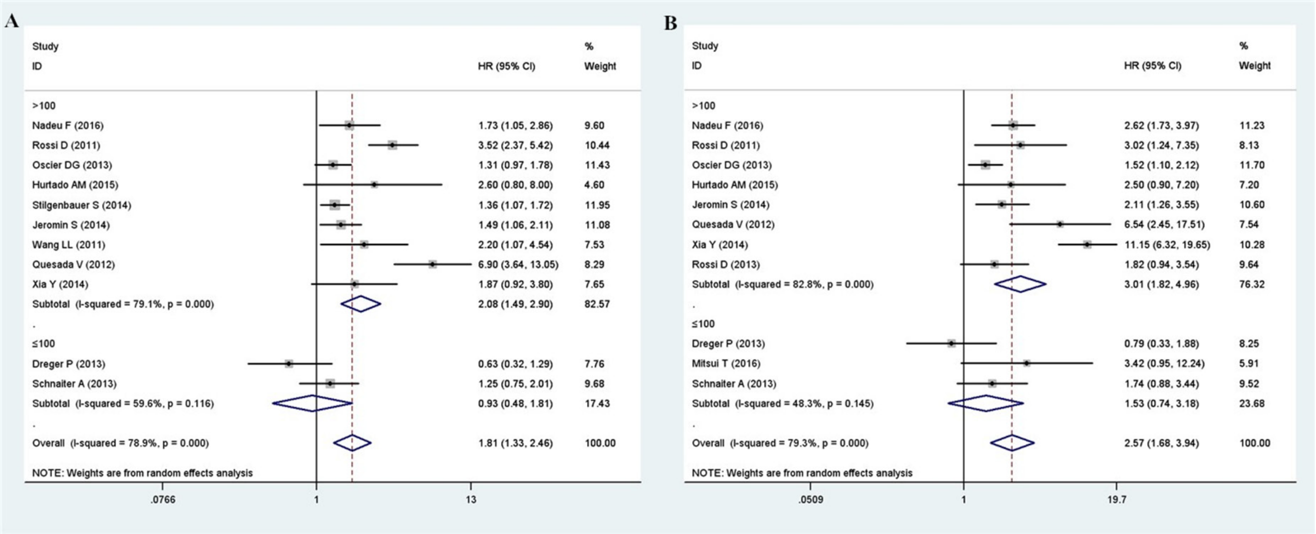

Supplementary Figure 2: Sample size subgroup analysis of the association between SF3B1 mutation and PFS **(A)** / OS **(B)** of CLL.

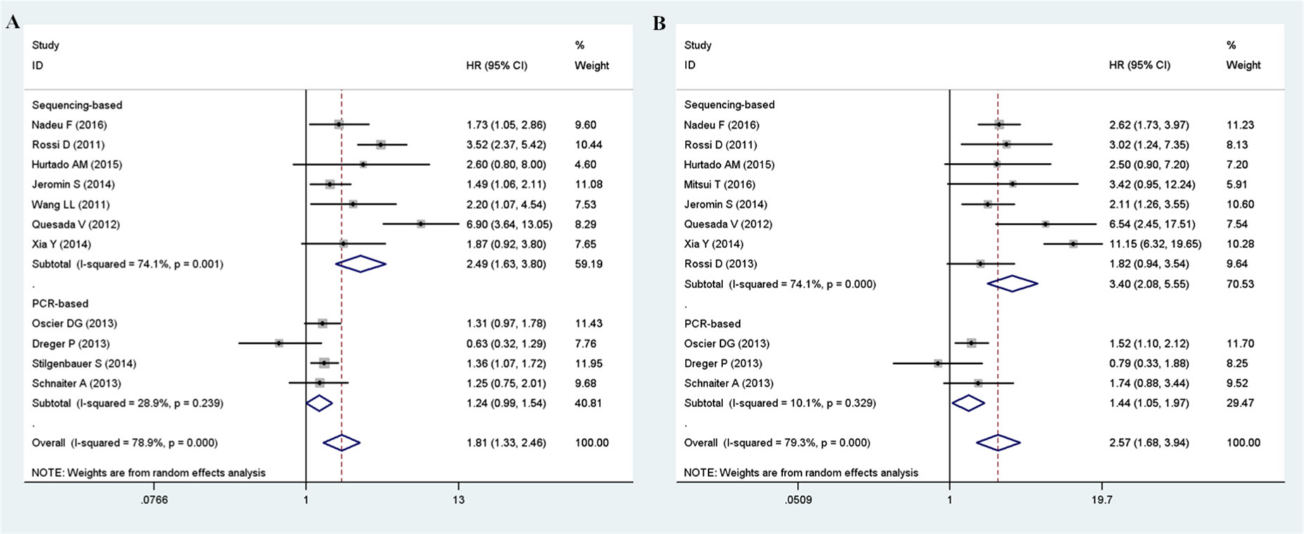

Supplementary Figure 3: Detection methods subgroup analysis of the association between SF3B1 mutation and PFS **(A)** / OS **(B)** of CLL.

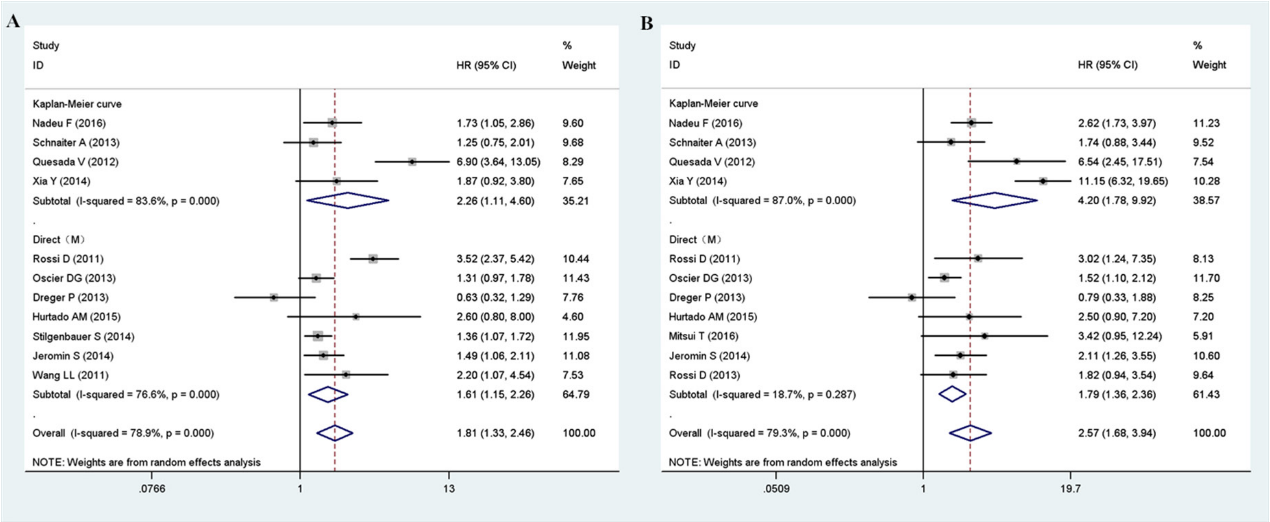

Supplementary Figure 4: Data extraction subgroup analysis of the association between SF3B1 mutation and PFS **(A)** / OS **(B)** of CLL.
